# Supplementary figures and images for: Imp interacts with Lin28 to regulate adult stem cell proliferation in the Drosophila intestine
Source: PLoS Genet. 2022 Sep 7;18(9):e1010385. doi: 10.1371/journal.pgen.1010385 (PMC9484684; doi:10.1371/journal.pgen.1010385)

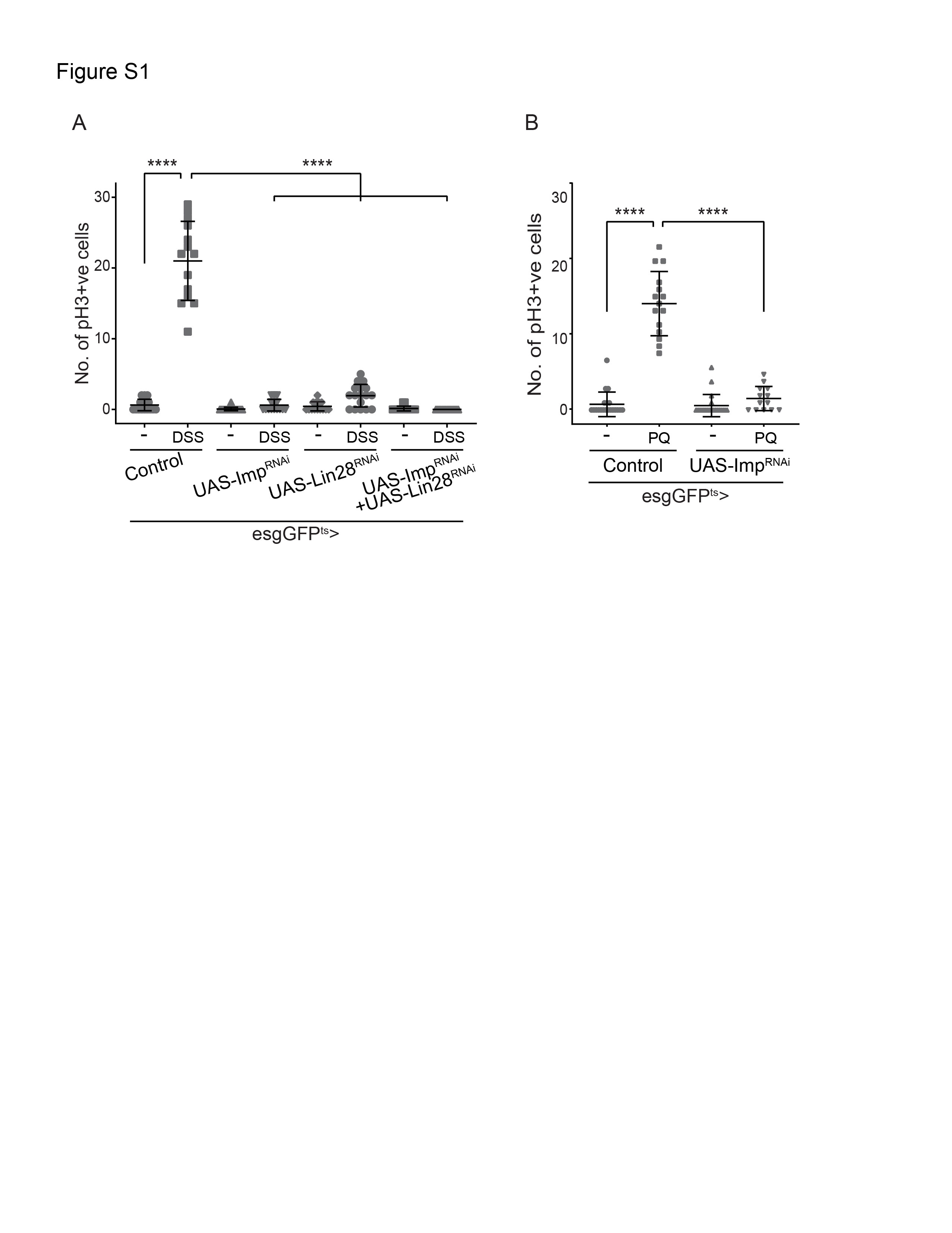

Supplement: S1 Fig — (A) Plot depicting the quantification of the number of pH3 positive cells in guts with esg-specific knockdown of Imp, Lin28, or Imp and Lin28 following 48-hours DSS treatment. Single perturbations, as well as double knock-down, result in almost complete block of cell proliferation. (B) Plot depicting the quantification of proliferation as measured by the number of phospho-Histone3 (pH3) positive cells per gut in control vs esg>ImpRNAi after 24-hour exposure to paraquat. In both panels, each data point represents the number of pH3 positive cells per gut. Student’s t-test **** p-value = 0.0001. (JPG) [file pgen.1010385.s001.jpg]

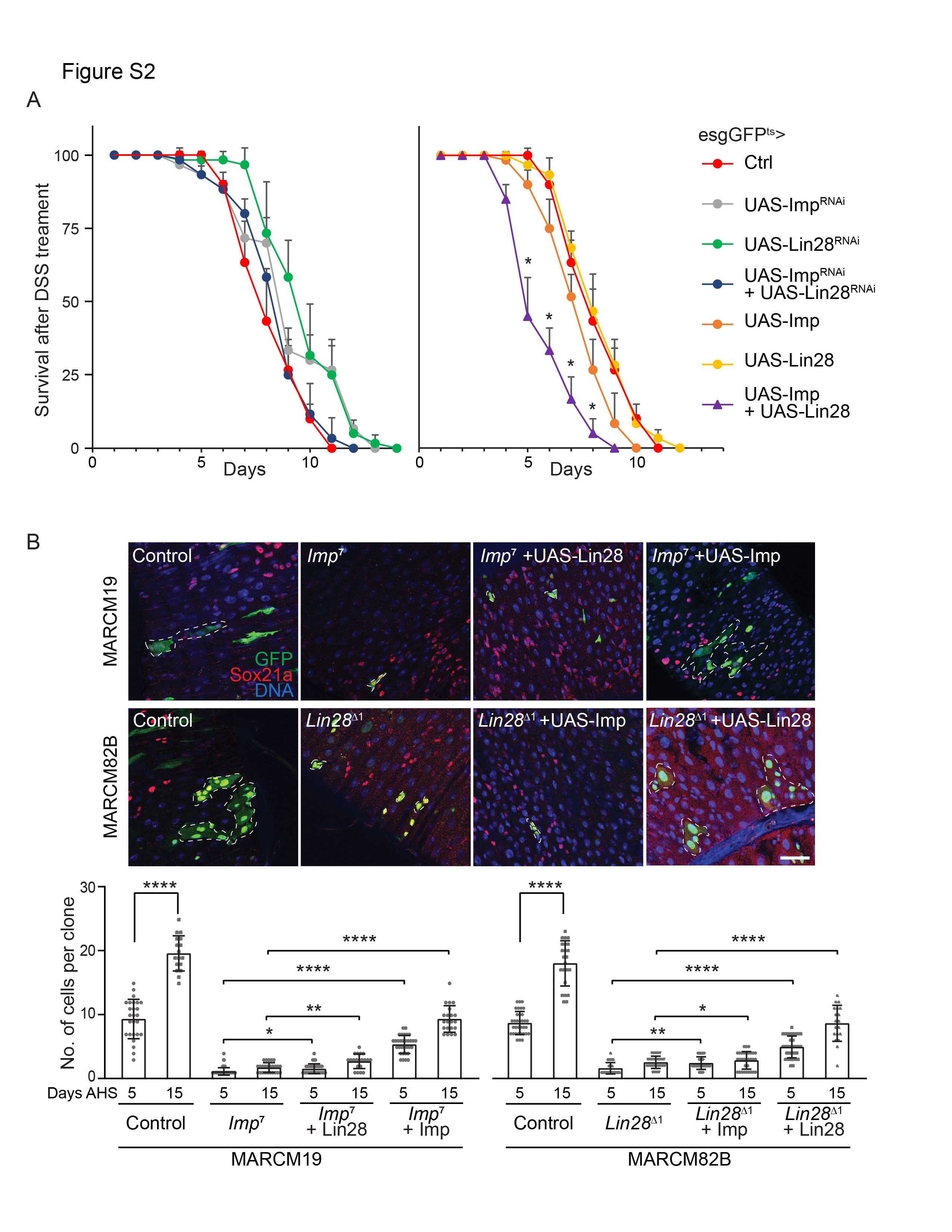

Supplement: S2 Fig — (A) Survival after exposure to DSS. Curves represent that average and standard deviation of 3 populations of 20 flies per conditions. Note that the same control is presented in both panels and curve are presented in 2 groups for better clarity. Simultaneous over-expression of Imp and Lin28 results in significantly greater sensitivity to DSS, while other manipulation does not affect survival under these conditions. Student’s t-test * p-value < 0.05 compared to controls at these timepoints. (B) Imp and Lin28 are not genetically redundant in ISCs. Representative MARCM images show that Imp7 and Lin28Δ1 clones fail to grow, while over-expression of Imp and Lin28 respectively, restores significant clone growth. However, over-expression of Lin28 in Imp7 clones or over-expression of Imp in Lin28Δ1 clones does not rescue the proliferation blockage. The number of cells per clones in the different genetic backgrounds, 5 and 15 days after heat shock induction (AHS), are shown below. Student’s t-test **** p-value = <0.0001; ns = > 0.05. Scale bar: 10μm. (JPG) [file pgen.1010385.s002.jpg]

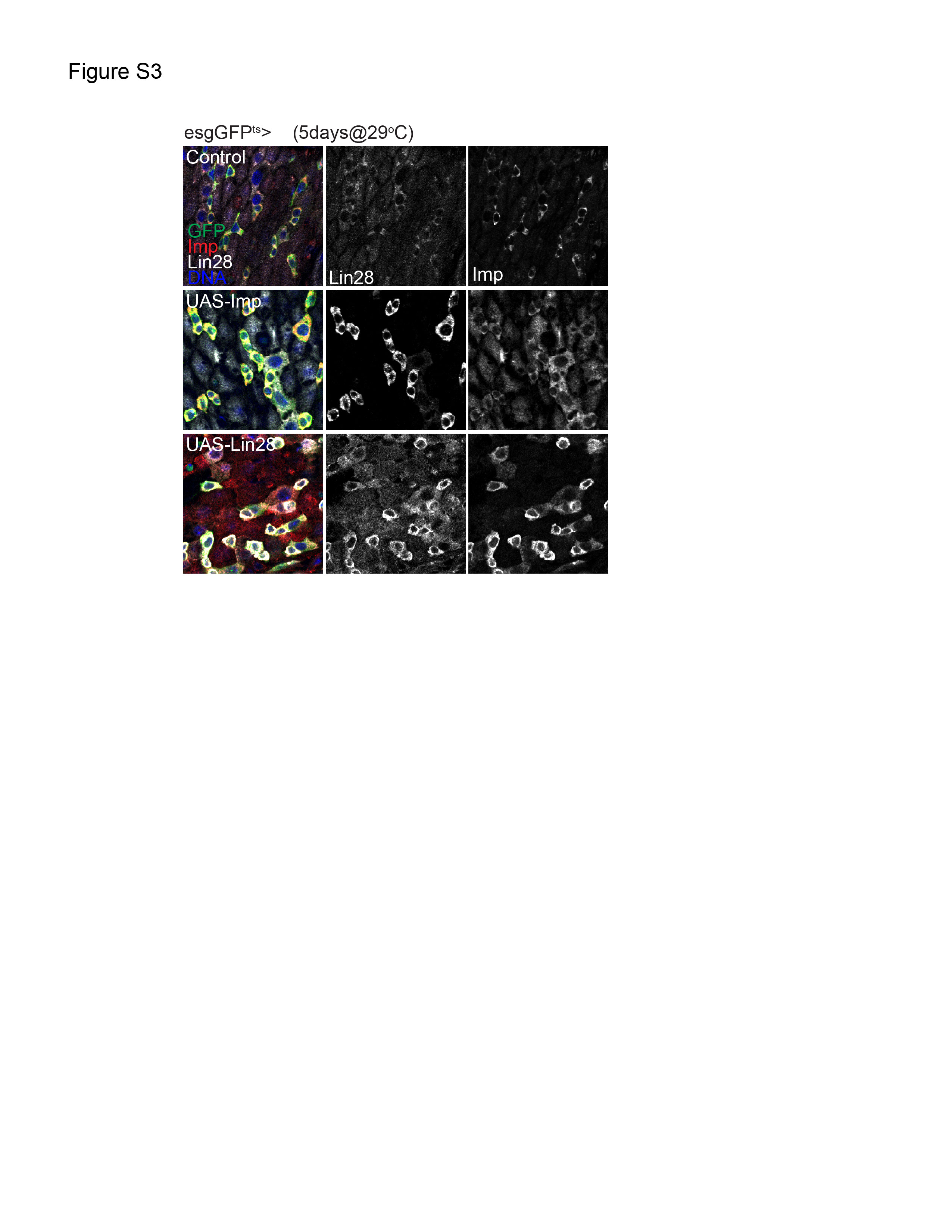

Supplement: S3 Fig — Representative confocal images show that elevated Imp and Lin28 proteins can both be detected in ISC/EBs of esgGFPts>UAS-Imp or esgGFPts>UAS-Lin28 animals, 5 days after transgene induction at 29°C. GFP (green) marks intestinal progenitors. DNA is stained with Hoechst. (JPG) [file pgen.1010385.s003.jpg]

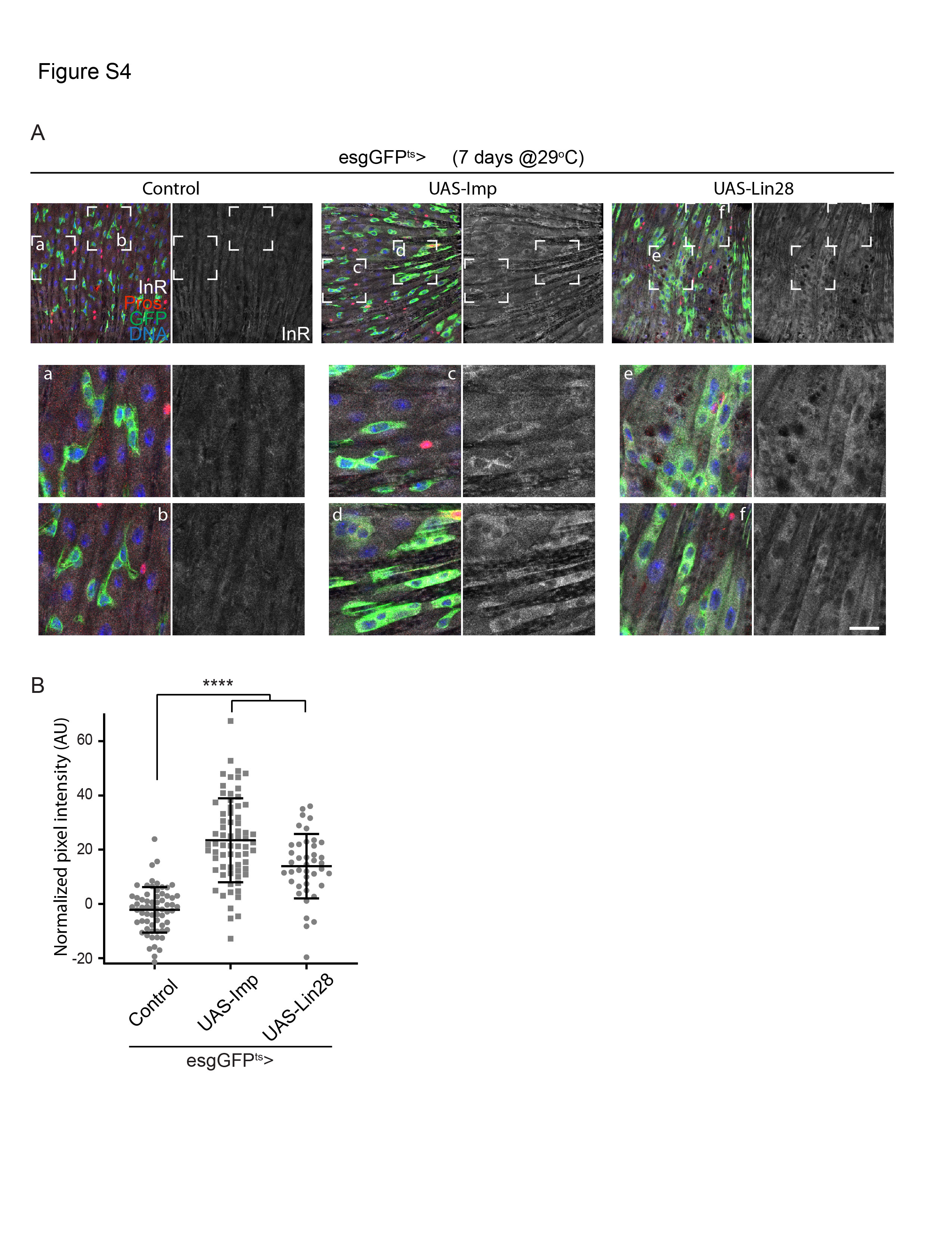

Supplement: S4 Fig — (A) Representative confocal images showing that over-expressing Imp for 7 days at 29°C in the ISCs and EBs leads to elevated expression of InR protein. GFP expression represents escargot positive cells (ISCs and EBs), Prospero stains for enteroendocrine cells, and Hoechst for DNA. Scale bar: 10μm. B) Quantification of the expression of InR protein in the esg-positive progenitors using Fiji/ImageJ shows that there is a significant elevated expression of InR in cells over-expressing either Imp or Lin28. Each data point represents individual cells quantified. (JPG) [file pgen.1010385.s004.jpg]

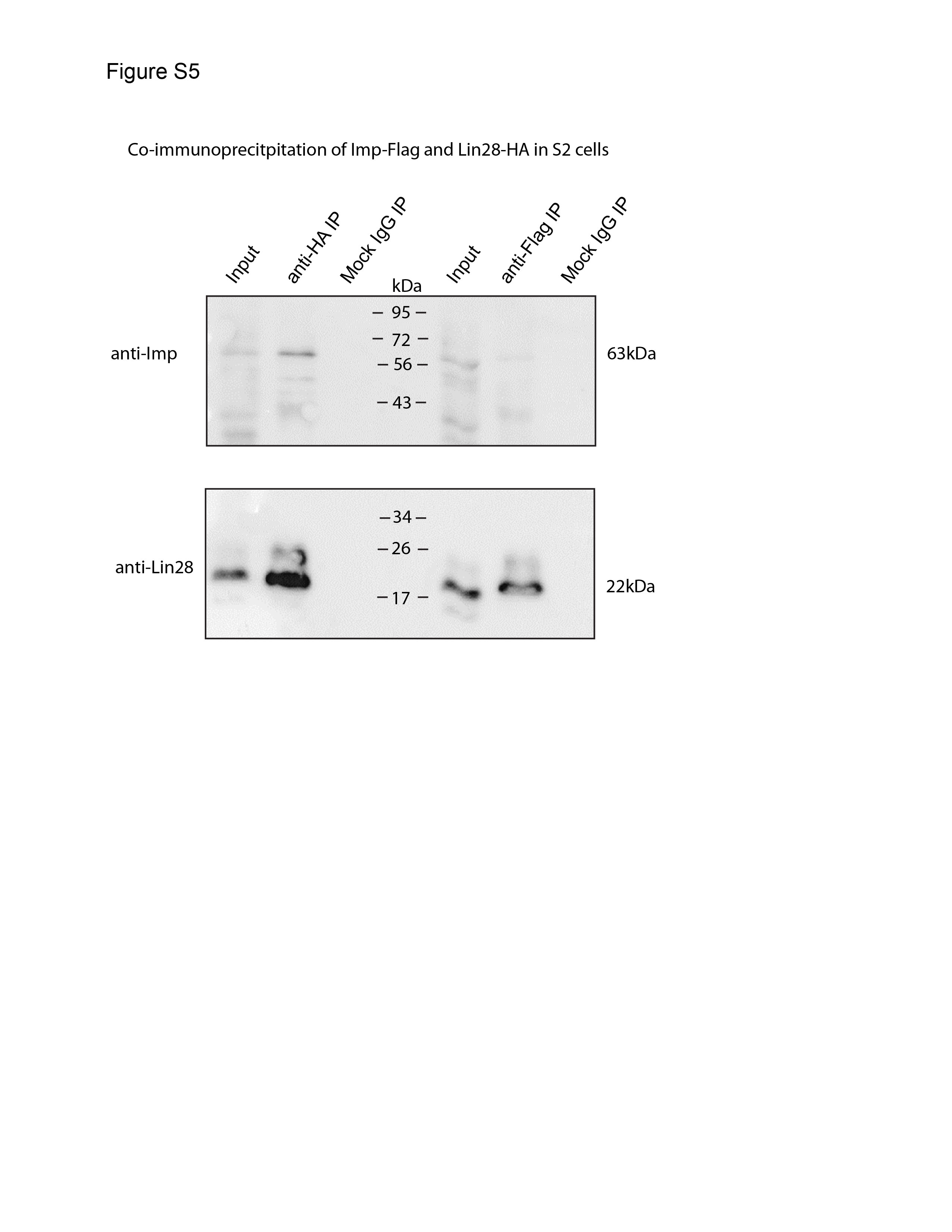

Supplement: S5 Fig — Western blot showing the reciprocal co-immunoprecipitation of Imp and lin28 using Flag-tagged Imp and HA-tagged Lin28 co-transfected in S2 cells. Inputs show expression of the fusion proteins, and immunoprecipitation using a non-specific IgG serve as controls. (JPG) [file pgen.1010385.s005.jpg]

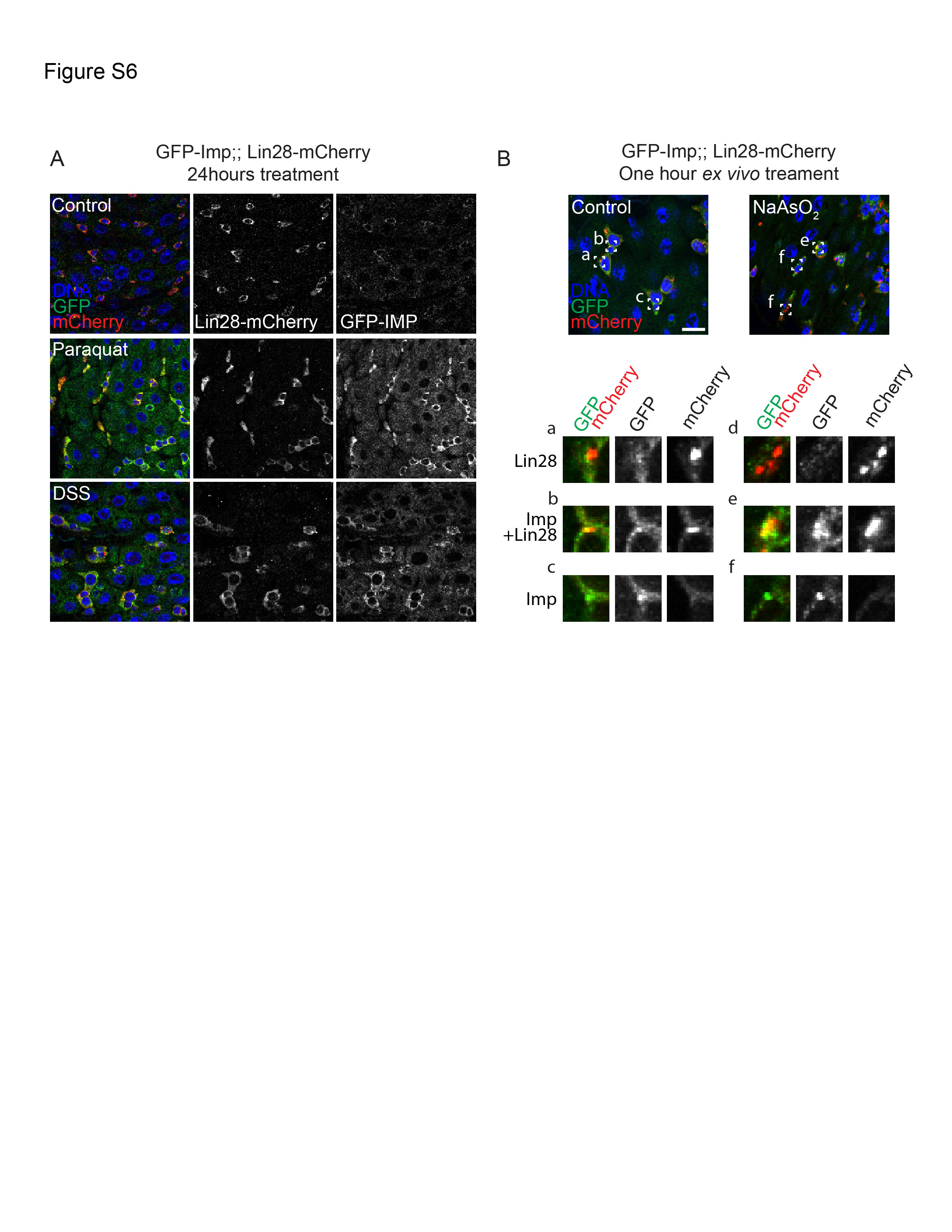

Supplement: S6 Fig — (A) Representative confocal images showing the expression of GFP-Imp (green) and Lin28-mCherry (red) under control conditions and in response to DSS or paraquat for 24 hours. In all conditions, the two fusion proteins can be detected in partially overlapping pattern in small diploid intestinal progenitors. DNA is stained with Hoechst. (B) Representative confocal images showing the expression of GFP-Imp (green) and Lin28-mCherry (red) after ex vivo treatment with Sodium Arsenite for 1 hour. Inserts a-f present higher magnification images of Imp, Lin28 and Imp+lin28 foci. Both under control conditions and after NaAsO2, all three types of granules can be detected in intestinal progenitors. DNA is stained with Hoechst. Scale bar: 3μm. (JPG) [file pgen.1010385.s006.jpg]
