# Supplementary material for: Imp interacts with Lin28 to regulate adult stem cell proliferation in the Drosophila intestine
Source: PLoS Genet. 2022 Sep 7;18(9):e1010385. doi: 10.1371/journal.pgen.1010385 (PMC9484684; doi:10.1371/journal.pgen.1010385)
Supplement: Attachment — Submitted filename: Sreejith_Imp_ResponseToReviewer.pdf [file pgen.1010385.s008.pdf]

Firstly, we would like to thank the reviewers for their comments and suggestions. We have now conducted additional experiments and edited our manuscript to address all the points raised. We believe that their comments helped us significantly improve our manuscript. The newly presented data fully support our conclusion that Imp cooperate with Lin28 to control ISC proliferation and intestinal homeostasis.

All major revision and additions are highlighted in Red in the annotated version of the revised manuscript.

We are directly addressing each of the specific points below.

Reviewer #1: In the Sreejith et al manuscript the authors set out to explore how Imp and Lin28 might corporate to intestinal stem cell (ISC) proliferation and cell fate. Studies have shown that Lin28 plays a key role in progenitor cells by promoting food-induced symmetric cell divisions through posttranscriptional stabilization of the InR mRNA. Furthermore, previous studies suggest that fly Lin28 and Imp might cooperate to stabilize Upd mRNA in the hub (niche) cells of the testis to promote stem cell renewal. The authors therefore set out to explore whether Imp and Lin28 might cooperate in adult ISCs to promote their divisions. They show that Imp is expressed in progenitor cells and, like Lin28, is required in the ISCs to promote their divisions in both homeostatic conditions and following tissue damage. They further show that although Lin28 and Imp can promote ISC divisions on their own, overexpression of both strongly enhances short-term effects on ISC proliferation. This leads the authors to propose that Lin28 and Imp act in a cooperative manner. Consistent with this, knockdown of Lin28 or Imp in the ISCs slightly reduces lifespan, while knockdown of both strongly reduces lifespan. They then show that Imp, like Lin28, binds the InR mRNA, and that OE of both Lin28 and Imp increases InR mRNA levels much more than OE of them on their own, suggesting that they cooperate to stabilize InR levels. Finally, they use different approaches to show that Lin28 and Imp interact directly in vitro and in vivo. While their in vitro data are very solid and suggest that Lin28 and Imp interact directly, the in vivo data are less convincing. All in all, this reviewer find that the majority of the data presented are very solid and that story is of interest to the audience of plus genetics when the following additions:

Major points:

1) Imp expression is supposed to be stress-responsive, suggesting that it might integrate environmental stimuli related to tissue damage. Does loss of Imp reduce survival following DSS- or paraquat-induced tissue damage?

We conducted stress survival experiments following DSS treatment. These data confirm that the co-overexpression of Imp and Lin28 using the esgGal4ts>GFP driver renders flies sensitive to tissue damage. Conversely, as requested by the reviewer, we found that loss of Imp and/or Lin28 in esg-positive cells is not sufficient to cause a significant phenotype in this short-term experiment.

These data are presented in Fig S2A and described in our revised manuscript.

It is interesting to note that loss of ets21c, which controls Imp expression, is also not associated with increased stress sensitivity (see Mundorf et al. 2019). Similarly, we have previously observed that short-term block of proliferation (e.g. by manipulation EGFR pathway to Sox21a expression) does not systematically results in increase sensitivity to acute stress.

2) In figure 4C the authors show that ubiquitous knockdown of either Imp or Lin28 reduces InR levels in the gut. Does this take into account that the number of ISCs are reduced in these conditions (after 7 days)? It would probably be more accurate to measure InR protein levels in ISCs by immunostaining in this condition – if feasible?

We have now included immunostaining showing the strong loss of InR protein in Delta-positive ISCs in both *Imp7* and *Lin28* mutants. This confirms our qRT-PCR results. These new data are presented in Fig4D.

3) The authors show that Lin28 and Imp localize in foci that are partially overlapping (Fig. 6B). This reviewer wonders if there is an increase in the overlap between Lin28 and Imp positive loci in conditions where symmetric ISC divisions are favored (either during regenerative growth or in the context of starvation-refeeding)?

We now include additional data to answer the reviewer's question. We now include high magnification images of immunostaining against endogenous Imp and Lin28 proteins, showing the partial overlap in signals. In addition, we found that in response to DSS, paraquat or NaAsNO2, Imp and Lin28 proteins remain in partially overlapping foci. This supports the notion that these 2 factors exist in common and specific RNPs.
These new data are presented in Fig6B and FigS6A,B.

4) This reviewer is not convinced about the Co-IP presented in figure 6C. It is not clear which of the bands correspond to Imp-GFP or Lin28. It would be better to improve it or remove it.

In response to this comment and the reviewer2's comment, we have decided to remove our data presenting Co-IP between Imp and Lin28 proteins from intestinal extracts.
In our hands, the Imp protein is particularly susceptible to degradation, unless using detergent concentrations incompatible with Co-IP. In combination with the very high level of protease activity in the intestine, and despite all our efforts, we were not able to generate better quality data to demonstrate the ability of Imp and Lin28 to co-precipitate in intestinal extracts.
We believe that our in vitro interaction data and our improved co-localization data in vivo allow us to suggest that Imp and Lin28 interact in ISCs, but we understand the limitation of these approaches.

Minor point:

line 208: I believe that the figure reference should be 3A?

This is correct, Figure 3A is now correctly cited there.

Reviewer #2: This manuscript describes an association between Imp and Lin-28 that control intestinal stem cell regeneration. The results are novel, well described and of interest to the broader field of stem cell biology.
I have a few comments that I would like the authors to address:

Lines 137-138 state "Imp7 137 homozygous mutant clones remained constantly small, mostly single Dl-positive Sox21a-positive ISCs". It is very difficult to observe Dl+ Sox21a+ cells in the Imp7 mutant panel. Perhaps separation of channels or an insert at higher magnification would assist the reader.

We included a higher magnification image of the Dl/Pros and Sox21a channels in Figure2A to better illustrate our statement that imp mutant clones are small and contain DL+/Sox21a+ ISCs that fail to proliferate.

Lines 155-156 state "This supports the notion that it acts downstream of the stress-responsive transcription factor ets21c in these cells". What data allow the authors to make this statement? Is there already a genetic association between Imp and ets21c?

This statement refers to Mundorf et al. (Cell Reports 2019) that show that Imp expression is up-regulated downstream of JNK and ets21c and that Imp is required for ets21c over-expression mediated ISC proliferation. This reference is cited at the end of this sentence.

Could an example of the PH3 staining please be shown alongside the graph in Fig 2C.

We included images of the pH3 staining in Figure 2D, to illustrate the large increase in the number of pH3+ mitotic cells present in the intestinal epithelium of ISC-GFPts>Imp animals.

Lines 178-180 state "However, no significant rescue was observed when Lin28 was expressed in Imp null mutant clones or, conversely, when Imp was expressed in Lin28 null clones (Fig3B)"
The data are in fact significant, so perhaps reword to "very slight rescue".

This is right. We corrected our statement to mention the very slight rescue of Imp mutation by Lin28 and, conversely, of Lin28 mutant by Imp.

Could the authors please explain why in Fig 4A the effect of UAS-InR is the same as UAS-InR[ACT]. Is InR maximally stimulated in the midgut?

We have shown that expression of UAS-InR[wt] is sufficient to drive stem cell proliferation (Biteau, Karpac et al. PLOS Genetics 20210). In addition, we and others have shown that ISC respond to Insulin like peptides (dilps) originating from brain Insulin Producing cells (e.g. dilp2) as well as locally produced dilps (e.g. dilp3 in gut visceral muscle).
Based on these previous data, we thus conclude that, even under normal conditions, dilps gut concentration is not limiting and that over-expressed InR becomes activated – mimicking the expression of InR[act].

I found the relationship between InR mRNA and protein somewhat confusing. Upregulated mRNA was only observed when both Imp and Lin28 were expressed yet upregulated InR protein was observed with either UAS-Imp or UAS-Lin28. Can the authors please offer an explanation.

The main difference between these experiments is the induction time (which we forgot to indicate in the FigS3).
In Figure 4B, we induced the expression of each transgene or the combination for 2 days at 29ºC, to prevent the expansion of the esg-positive population (as seen in Fig3A) and allow us to normalize the InR mRNA qPCR signal.
In Figure S3, we induced the expression of UAS-Imp and UAS-Lin28 for 7 days to allow InR protein to accumulate and levels rise above background staining.
Figure3 and its legend now mention the induction time. The description of these data in the result section has been updated too.

The Co-IP of Lin28 with IMP is convincing in Fig 5B but the anti-Imp western looks like a smear. Is the protein easily degraded? The same is seen in Fig 6C. Could a comment please be made about these westerns and the expected sizes of the protein bands.

As detailed in our response to reviewer1's comments on the Co-IP, the Imp protein is indeed particularly sensitive to degradation in our hands. We indicated protein sizes in Fig5B. Because of these technical limitations, we also have decided to remove the Co-IP data from intestinal extracts.
We believe our additional data demonstrating the partial co-localization of Imp and Lin28 in intestinal progenitors, combined with our in vitro interaction data, allow us to suggest that Imp and Lin28 likely interact in intestinal cells.

Minor comments:
Line 139 – I think this should refer to Fig 2A, not Fig (2B and 2C)

We corrected this.

Line 150 – Please define the ISC-Gal4. Is this Delta-Gal4?

We agree this was unclear and apologize for the confusion. The ISC-specific driver we refer to as 'ISC-Gal4ts' is esgGal4,UAS-YFP,tubGal80ts;Su(H)Gal80 originally from S.Hou and used in the Jasper lab in numerous studies.
We more explicitly mention this in the manuscript and indicate the full driver genotypes that were missing in Material & Methods section.

Reviewer #3: Post-transcriptional regulation has emerged as a main regulatory level involved in the control of stem cell proliferation. Among the RNA binding proteins found to play a conserved role in this process are Lin-28 and Imp/IGF2BP. These two proteins have been shown to cooperate for the control of stem cell proliferation in different contexts, in both mammalian and fly systems.
In this manuscript, the authors aimed at investigating if Drosophila Imp is required for the control of Intestinal Stem Cell (ISC) proliferation. As Lin-28 was already known to control this process through the regulation of InR mRNA, which encodes the Insulin receptor, they investigated whether Lin-28 and Imp could cooperate to regulate InR expression.
Combining functional studies and biochemical binding assays, they convincingly show that Lin-28 and Imp cooperate to promote the proliferation of ISCs in vivo and that they interact directly. Furthermore, they show that Imp, similarly to Lin-28, binds to InR mRNA and promotes ISC proliferation by promoting the expression of InR.
That Lin-28 and Imp cooperate for the control of stem cell proliferation is not really novel, but the authors provide an additional strong example that will likely be of interest both for the community of researchers working on intestinal homeostasis as well as for RNA biologists. Although the resolution of the provided Figures was limited, the presented data appear to be of high quality. Furthermore, the authors have combined various complementary approaches to support their conclusions.
Beyond minor points listed below, my major point relates to the lack of information related to the regulation of Imp and Lin-28 expression, which would nicely complement the functional and biochemical assays and help understand the regulatory process under study.

Major point:
The authors describe the expression of Imp and Lin-28 in wild-type contexts, but did not investigate 1) if the two proteins are regulating each other, and 2) if their expression is induced in response to stress.

We agree that additional data regarding the regulation of Imp and Lin28 expression in ISCs and EBs improves our understanding of the role of these two factors in tissue homeostasis and regeneration.

We now show that the over-expression of Imp using the esgGFPts driver cause increased Lin28 levels in esg-positive cells, and that conversely, Lin28 over-expression induces Imp protein expression in these cells. These data are reported in the new FigS3.

Regarding the stress responsiveness of Imp and Lin28, it has been previously reported that Lin28 levels increase in response to starvation (Chen ... and Sokol, Development 2015 and Luhur and Sokol, Fly(Auston) 2015), and that Imp is induced by the activation of the JNK pathway (Mundorf ... and Uhlirova, Cell Reports 2019). In the stress conditions that we used to investigate the role of Imp in controlling ISC proliferation, DSS and paraquat, we now show that treatment with these compounds leads to a detectable change in GFP-Imp expression, bit no obvious change in Lin28-mCherry levels. These data are now presented in the new FigS6A.

Addressing point 1 would help interpret the genetic interactions observed in vivo and can easily be done by checking the expression of Imp in Lin-28 mutant and overexpression contexts (and vice versa). Addressing point 2 would help understand whether chemical stress (exposure to DSS) induces proliferation by promoting the expression of Imp and Lin-28.

Minor points:
1) The authors should better describe the different populations of cells found in the intestine (EE is never defined and EB is defined only after the first time it is used).

We now define the ISC lineage and the different cell type in the introduction.

2) why the authors wonder if Lin-28 and Imp act redundantly (line 175) is not clear, as mutating either of them produces a very strong phenotype. Why they thought expressing Lin-28 would rescue the imp phenotype (and vice-versa) is also not clear. Replacing Figure 3B by Supplementary Figure S2B would better illustrate functional cooperativity.

We agree with this suggestion and have now combined the double gain-of-function and double loos-of-function in Fig3. We moved the rescue experiments to FigS2, as these do not directly test the notion that Imp andLin28 cooperate.

3) The authors refer to the Imp protein-trap line as Imp-GFP. As the GFP is fused in frame N-terminally, it should rather be termed GFP-Imp (both in the text and in Figures).

This is a good suggestion; we have switch to GFP-Imp to better describe the trap line.

4) Figure 4D: the authors should better explained how normalization of qPCR data has been performed.

We now indicate more clearly that the rp49 expression was used to normalize these data in the figure legend. Following the suggestion made by Reviewer1, we now added immunostaining data showing reduced InR levels in Imp and Lin28 mutants, supporting the qRT-PCR and our interpretation that loss of Imp or Lin28 results in decreased InR levels in ISCs and EBs.

5) Figure 6B: the arrows in a-d are not positioned correctly (the don't point to granules under study)

In this figure, the arrows do not point to specific granules. They represent the 5micron long lines that were used to generate the intensity profiles for GFP and mCherry signal. We modify slightly the figure legend to clarify this further.

6) line 285: the IP-RT-QPCR performed is NOT indicative of direct binding to InR mRNA.

We agree and have updated this sentence.

7) The authors should follow the nomenclature used in flies, ie use capitals for proteins only and italics for transcripts, genes and alleles.

We have corrected this in numerous instances.

8) a number of typos are left in the text

We have corrected several typos across the manuscript and the figures.

9) Indicating the nature of the statistical tests used in the Figure legends would be helpful.

We now mention the tests used.
