# Supplementary material for: Imp interacts with Lin28 to regulate adult stem cell proliferation in the Drosophila intestine
Source: PLoS Genet. 2022 Sep 7;18(9):e1010385. doi: 10.1371/journal.pgen.1010385 (PMC9484684; doi:10.1371/journal.pgen.1010385)
Supplement: Attachment — Submitted filename: Reviewer 1 comments on revised manuscript.docx [file pgen.1010385.s009.docx]

**Reviewer #1:**

This reviewer is generally satisfied with the revised version of the manuscript. However, the authors should discuss the point that they do not see an effect of Imp and Lin28 knockdown on the sensitivity of these flies to DSS (Response to Major point 1, see below)

In the Sreejith et al manuscript the authors set out to explore how Imp and

Lin28 might corporate to intestinal stem cell (ISC) proliferation and cell fate. Studies have

shown that Lin28 plays a key role in progenitor cells by promoting food-induced symmetric

cell divisions through posttranscriptional stabilization of the InR mRNA. Furthermore,

previous studies suggest that fly Lin28 and Imp might cooperate to stabilize Upd mRNA in

the hub (niche) cells of the testis to promote stem cell renewal. The authors therefore set

out to explore whether Imp and Lin28 might cooperate in adult ISCs to promote their

divisions. They show that Imp is expressed in progenitor cells and, like Lin28, is required in

the ISCs to promote their divisions in both homeostatic conditions and following tissue

damage. They further show that although Lin28 and Imp can promote ISC divisions on their

own, overexpression of both strongly enhances short-term effects on ISC proliferation. This

leads the authors to propose that Lin28 and Imp act in a cooperative manner. Consistent

with this, knockdown of Lin28 or Imp in the ISCs slightly reduces lifespan, while knockdown

of both strongly reduces lifespan. They then show that Imp, like Lin28, binds the InR mRNA,

and that OE of both Lin28 and Imp increases InR mRNA levels much more than OE of them

on their own, suggesting that they cooperate to stabilize InR levels. Finally, they use

different approaches to show that Lin28 and Imp interact directly in vitro and in vivo. While

their in vitro data are very solid and suggest that Lin28 and Imp interact directly, the in vivo

data are less convincing. All in all, this reviewer find that the majority of the data presented

are very solid and that story is of interest to the audience of plus genetics when the

following additions:

Major points:

1) Imp expression is supposed to be stress-responsive, suggesting that it might integrate

environmental stimuli related to tissue damage. Does loss of Imp reduce survival following

DSS- or paraquat-induced tissue damage?

We conducted stress survival experiments following DSS treatment. These data confirm

that the co-overexpression of Imp and Lin28 using the esgGal4ts>GFP driver renders flies

sensitive to tissue damage. Conversely, as requested by the reviewer, we found that loss of

Imp and/or Lin28 in esg-positive cells is not sufficient to cause a significant phenotype in

this short-term experiment.

**These data are presented in Fig S2A and described in our revised manuscript.**

**These data should be discussed in the main text**

It is interesting to note that loss of ets21c, which controls Imp expression, is also not

associated with increased stress sensitivity (see Mundorf et al. 2019). Similarly, we have

previously observed that short-term block of proliferation (e.g. by manipulation EGFR

pathway to Sox21a expression) does not systematically results in increase sensitivity to

acute stress.
