# Supplementary material for: Imp interacts with Lin28 to regulate adult stem cell proliferation in the Drosophila intestine
Source: PLoS Genet. 2022 Sep 7;18(9):e1010385. doi: 10.1371/journal.pgen.1010385 (PMC9484684; doi:10.1371/journal.pgen.1010385)
Supplement: Attachment — Submitted filename: Reviewers_Response_R2.docx [file pgen.1010385.s010.docx]

We would like to thank the reviewers for their additional comments.

In this second revision, we have addressed the remaining issues: (i) We have fixed the incorrect figure reference, (ii) edited the cited paragraph title, and (iii) added a sentence to discuss the data presented in S2A and the absence of sensitivity to DSS of the IMP and Lin28 knockdown flies.
